# Exposure to Large-Scale Social and Behavior Change Communication Interventions Is Associated with Improvements in Infant and Young Child Feeding Practices in Ethiopia

**DOI:** 10.1371/journal.pone.0164800

**Published:** 2016-10-18

**Authors:** Sunny S. Kim, Rahul Rawat, Edina M. Mwangi, Roman Tesfaye, Yewelsew Abebe, Jean Baker, Edward A. Frongillo, Marie T. Ruel, Purnima Menon

**Affiliations:** 1 Poverty, Health and Nutrition Division, International Food Policy Research Institute, Washington, D.C., United States of America; 2 Poverty, Health, and Nutrition Division, International Food Policy Research Institute, Dakar, Senegal; 3 Poverty, Health, and Nutrition Division, International Food Policy Research Institute, Addis Ababa, Ethiopia; 4 Alive & Thrive, FHI 360, Addis Ababa, Ethiopia; 5 Alive & Thrive, FHI 360, Washington, D.C., United States of America; 6 Department of Health Promotion, Education, and Behavior, University of South Carolina, Columbia, SC, United States of America; 7 Poverty, Health, and Nutrition Division, International Food Policy Research Institute, New Delhi, India; University College London, UNITED KINGDOM

## Abstract

Optimal breastfeeding (BF) practices in Ethiopia are far below the government’s targets, and complementary feeding practices are poor. The Alive & Thrive initiative aimed to improve infant and young child feeding (IYCF) practices through large-scale implementation of social and behavior change communication interventions in four regions of Ethiopia. The study assessed the effects of the interventions on IYCF practices and anthropometry over time in two regions–Southern Nations, Nationalities and Peoples Region and Tigray. A pre- and post-intervention adequacy evaluation design was used; repeated cross-sectional surveys of households with children aged 0–23.9 mo (n = 1481 and n = 1494) and with children aged 24–59.9 mo (n = 1481 and n = 1475) were conducted at baseline (2010) and endline (2014), respectively. Differences in outcomes over time were estimated using regression models, accounting for clustering and covariates. Plausibility analyses included tracing recall of key messages and promoted foods and dose-response analyses. We observed improvements in most WHO-recommended IYCF indicators. Early BF initiation and exclusive BF increased by 13.7 and 9.4 percentage points (pp), respectively. Differences for timely introduction of complementary foods, minimum dietary diversity (MDD), minimum meal frequency (MMF), minimum acceptable diet (MAD), and consumption of iron-rich foods were 22.2, 3.3, 26.2, 3.5, and 2.7 pp, respectively. Timely introduction and intake of foods promoted by the interventions improved significantly, but anthropometric outcomes did not. We also observed a dose-response association between health post visits and early initiation of BF (OR: 1.8); higher numbers of home visits by community volunteers and key messages recalled were associated with 1.8–4.4 times greater odds of achieving MDD, MMF, and MAD, and higher numbers of radio spots heard were associated with 3 times greater odds of achieving MDD and MAD. The interventions were associated with plausible improvements in IYCF practices, but large gaps in improving children’s diets in Ethiopia remain, particularly during complementary feeding.

## Introduction

Despite being one of the poorest countries in the world with an estimated population of 97 million people predominantly living in rural areas [1], Ethiopia has made considerable progress in reducing infant, child, and maternal mortality over several decades by expanding primary health care services and improving the quality of health service provision through its Health Sector Development Program (HSDP) [2]. Child undernutrition remains high, however, with an estimated prevalence of stunting of 40% among children less than five years of age in 2014, down from 51% in 2005. Wasting is also highly prevalent, affecting 9% of children under five years, down from 12% in 2005 [3].

Adequate infant and young child feeding (IYCF) practices contribute to healthy child growth and development [4]. In Ethiopia, Demographic Health Survey data from 2011 indicated that several IYCF practices were sub-optimal, with only 52% of infants starting breastfeeding within one hour of birth (a decline from 69% in 2005) and 52% of children 0–6 months of age being exclusively breastfed (EBF) (a slight increase from 49% in 2005) [5]. These practices are far below the HSDP IV targets of increasing early initiation of BF to 92% and EBF among 0–6 months old children to 70% by 2015 [6]. Complementary feeding (CF) practices were also poor; only 49% of children 6–8 months of age had consumed any solid, semi-solid, or soft foods in the previous 24 hours, less than 5% of children 6–23 months of age had consumed at least four food groups (minimum dietary diversity), and 4% had achieved the minimally acceptable diet [5]. Thus, there is an urgent need to improve IYCF practices in Ethiopia, particularly through implementation of effective interventions at large scale.

Various approaches have been shown to be effective in improving IYCF practices. Systematic reviews of interventions such as individual- and group-based peer counseling and support provided by lay counselors or trained professionals have shown impacts on improving BF practices [7–9], but evidence of whether the magnitude of impacts observed can be achieved when programs operate at large-scale is lacking [10,11]. Studies looking at the promotion of optimal CF practices through the provision of nutrition education and/or food supplements have focused mainly on impacts on child anthropometry [12–15]; a systematic review found that education interventions improved height-for-age (HAZ) and weight-for-age z-scores (WAZ), reduced stunting, and resulted in a large increase in intake of recommended foods [15]. Many of the interventions included in these reviews and studies were efficacy studies or small-scale interventions which followed individual children over time. As a result, there is limited evidence of population-level impact from evaluations of large-scale programs showing what works to improve caregiver’s knowledge and practices related to CF, how these changes lead to positive child outcomes, and what factors enable successful scale-up of these interventions [16].

This paper presents findings from an evaluation of large-scale social and behavior change communication interventions aimed at improving IYCF practices and child anthropometric outcomes in Ethiopia.

### Program description

Alive & Thrive (A&T) Ethiopia is a multi-year initiative that started in 2009 and aimed at reducing undernutrition caused by suboptimal BF and CF practices [17]. The program adopted a four-component implementation framework to achieve scale: 1) advocacy and policy dialogues at the national and sub-national levels, 2) interpersonal communication (IPC) and community mobilization, 3) mass communication, and 4) strategic use of data. IPC focused on the delivery of seven key messages on IYCF to targeted mothers, and community mobilization and mass media interventions were directed at opinion leaders, fathers and other caregivers. At the community level, age-appropriate IYCF messages and counseling to mothers and caregivers of children less than two years of age were delivered primarily through the Federal Ministry of Health’s flagship Health Extension Program, utilizing the large network of government-salaried female health extension workers (HEWs) and cadres of community health volunteers known as the Women’s Development Armies (WDAs) or Health Development Armies (HDAs). Coverage of A&T community-based interventions were intended to be achieved at scale through different implementation partners in the four most populous regions—Amhara, Oromia; Southern Nations, Nationalities and Peoples Region (SNNPR); and Tigray. The partners included the USAID-funded Integrated Family Health Program, led by Pathfinder and JSI, region-specific nongovernmental organizations such as the Relief Society of Tigray (REST), and other local organizations such as faith-based organizations and women’s associations. In the communities, the HEWs and community volunteers, i.e., WDAs or HDAs, conducted IPC through counseling at the health post or during routine home visits and community mobilization activities such as village gatherings to discuss IYCF and food demonstrations (i.e., in preparation of enriched complementary foods). In addition to the community-based interventions, a mass media campaign was launched in the four regions to promote IYCF messages mainly through the radio. Detailed description and implementation process of the interventions are documented in a separate paper [18]. During the intervention period, the A&T model reached large scale with an estimated 1.5 million mothers of children under two years exposed to IPC across 295 intervention woredas (districts). An estimated 960,000 women heard the A&T radio spots, for an estimated total of 2 million mothers of children under two years reached by either IPC or mass media [19].

## Methods

### Evaluation design

The evaluation used an adequacy design based on before and after comparisons in two of the four program regions (SNNPR and Tigray), to determine whether changes in intended outcomes were observed and overall program objectives were met. An adequacy design draws inferences about the adequacy of program outcomes by comparing with previously established adequacy criteria or expected objectives; adequacy assessment requires no control groups [20]. This type of evaluation design does not allow causal inference, however, and for this reason, our paper does not report “impacts” on our key outcomes, but rather “associations” between program and outcomes. An adequacy design was used in this study due to the prior arrangements between the implementation partner and the Ethiopian health extension platform and the mandate to operate at scale [21].

A cross-sectional household survey was conducted at baseline (2010) and four years later (2014) among households with children 0–59.9 months of age; surveys were conducted in August-September during both years in order to minimize the effects of seasonality on outcomes of interest. The key outcomes were the WHO-recommended core IYCF indicators related to BF and CF (for children 0–24 months of age) and stunting in children 24–59.9 months of age. Although interventions were targeted to children less than two years of age, we examined the change on stunting in a population of children 24 months of age and older to measure the accrued impacts of these interventions in the community once the period of greatest potential benefit had concluded (i.e., time period of full intervention exposure during the critical window of opportunity between pregnancy and 24 months of age). In addition to the results of intervention adequacy, we present findings from plausibility analyses, including an analysis of changes in intermediary outcomes such as the recall of key messages and food items promoted by the interventions, and a dose-response analysis looking at outcomes by timing and intensity of intervention exposure.

### Sample size estimation and sampling method

Sample size calculations were made to detect differences in the primary outcomes, i.e., EBF rates among infants 0–5.9 months of age, CF practices among children 6–23.9 months of age, and stunting among children 24–59.9 months of age, between baseline and endline, assuming alpha of 0.05, power of 0.80, intra-class correlation (ICC) of 0.01 for clustering at the *woreda* (district) level (estimated from previous surveys), and estimated baseline prevalence of the primary outcomes. Assuming a baseline prevalence of 49% for EBF [22], we estimated a total sample of 600 infants aged 0–5.9 months was sufficient to detect at least an 8 percentage point (pp) difference in the proportion of children achieving EBF at endline. A total sample of 900 children aged 6–23.9 months was sufficient to detect at least a 6.5 pp difference in the proportion of children achieving minimum dietary diversity at endline, from a baseline prevalence of 33% [22]. Additionally, a total sample of 1500 children aged 24–59.9 months was sufficient to detect at least a 12 pp difference in stunting at endline, assuming a baseline prevalence of 46.5% [22]. Prior to conducting the endline survey, we verified our detectable effect sizes based on the original sample size, the actual baseline prevalence values of our outcome indicators, and the ICC from the baseline survey. Based on these parameters, our detectable effect size decreased to 7 pp for EBF, 4 pp for minimum dietary diversity, and 7 pp for stunting. No changes were made to the sample sizes at endline. The final sample sizes attained were 606 and 619 children aged 0–5.9 months, 875 and 875 children aged 6–23.9 months, and 1,481 and 1,475 children aged 24–59.9 months at baseline and endline respectively.

A two-stage cluster sampling method was applied at baseline and endline. For the first stage, the primary sampling unit (i.e., cluster) was the enumeration areas (EAs) from the 89 rural woredas where the Integrated Family Health Program operates in the two regions, creating the sampling frame. The Central Statistical Authority for the Demographic Health Survey in Ethiopia randomly selected 75 EAs using probability proportion to size (PPS) sampling in relation to the population of all the EAs within the sampling frame. All 75 EAs surveyed at baseline were included in the endline survey. Sample sizes were not estimated separately for each region, but rather per clusters which were spread over both regions. By design, the evaluation was not intended to account for regional differences in any indicators of interest, either at baseline or in changes over time. For the second stage, a complete household listing with the number of children residing in each household within each selected cluster was conducted. This list included identification of all eligible survey respondents (i.e., mothers of children 0–59.9 months of age) to form three sampling frames: children aged 0–5.9 months, 6–23.9 months, and 24–59.9 months. From each sampling frame, study subjects were selected using systematic random sampling, i.e. selecting an integer divisible by the sample size required for each sampling frame and less than the total number of eligible households, then starting with the household corresponding to that integer on the list and selecting the next households corresponding to the integers in progression at a constant difference. Households selected to participate for one age category were not included in the other sampling frames, even if they had children eligible for the other age groups.

### Outcome measures

The primary outcomes were IYCF practices using the WHO-recommended indicators for children 0–2 years of age [23] and the prevalence of stunting in children 24–59.9 months of age. The eight IYCF indicators examined were: 1) early initiation of BF, 2) EBF to six months, 3) continued BF at one year, 4) timely introduction of solid, semi-solid, or soft foods at 6–8.9 months, 5) minimum dietary diversity, 6) minimum meal frequency, 7) minimum acceptable diet, and 8) consumption of iron-rich or iron-fortified foods [24]. The IYCF indicators were constructed based on mothers’ previous-day recall about BF and of specific liquids and foods consumed by the study children. Anthropometric data were collected using standardized methods [25]. Weight and length/height were converted into height-for-age z-scores (HAZ), weight-for-age z-scores (WAZ), and weight-for-height z-scores (WHZ) according to the WHO child growth standards [26]. Stunting, underweight, and wasting were defined as <-2 z-score of HAZ, WAZ, and WHZ, respectively.

Two childhood illnesses (acute respiratory infection symptoms and diarrhea) were analyzed as secondary outcomes. The prevalence of symptoms of acute respiratory infection (ARI) was estimated by maternal recall of their children having been ill with a cough and shortness of breath in the two weeks preceding the survey. Mothers were also asked whether their children had diarrhea in the past two weeks.

### Intervention exposure and intensity measures

Social and behavior change communication (SBCC) interventions were delivered during home and health post visits, through the mass media (radio mainly), and through community mobilization activities in the form of community conversations and village gatherings. The latter included cooking demonstrations with locally available foods. Print materials, particularly the Child Nutrition Card (CNC) describing the seven recommended feeding behaviors (“Excellent Feeding Actions”), were developed for use in interpersonal and community sessions. Six radio spots were produced and aired, two on BF and four on CF. Thus, we defined intervention exposure as contact via these multiple SBCC channels: 1) IPC during health post visit, 2) IPC during home visit by HEW, 3) IPC during home visit by community volunteer, 4) CNC viewing, 5) group education during village gatherings or community conversations, 6) radio spots, and 7) community food demonstrations. Exposure to each channel within the last six months and intensity of exposure (number of contacts or messages/spots recalled) as well as to a combination of channels (number of channels exposed: low = 1 only, medium = 2–3, and high = 4 or more) were examined and used for dose-response analysis with each of the primary outcomes.

Data were also collected on maternal recall of specific promoted messages (in individual or group counseling, or mass media) to examine plausible links to the interventions. Examples include messages on the timely introduction of semi-solid foods starting at six months of age, meal frequency of at least three times a day, increasing dietary diversity by incorporating additional food groups in the child’s diet, and feeding the child specific foods such as thick porridge enriched with eggs, dried meat powder, and green leafy vegetables.

Timing of potential exposure to the interventions was also examined using child age at first exposure as the main criteria. First potential exposure after 24 months of age (beyond the first 1,000 days) was defined as “no exposure” (meaning, exposure to interventions likely occurred too late for the child to benefit in terms of IYCF practices and potential to benefit in terms of linear growth). “Late exposure” was defined as first potential exposure starting at ages 12–23.9 months, “early exposure” starting at ages 0–11.9 months, and “optimal timing of exposure” starting before birth (i.e. during pregnancy).

In addition to the A&T interventions, Community-based Nutrition (CBN), a government child nutrition program launched in 2008 [27], was present in many A&T intervention areas; 64% of the EAs at endline reported the presence of CBN in the communities. At the time of the study, CBN was focused on vitamin A supplementation, deworming, and screening and referral for moderately malnourished women and children under five years of age. Therefore, we examined the differences in outcomes and intervention exposure between CBN and non-CBN areas.

### Statistical analysis

Differences between baseline and endline were tested using linear regression models (for continuous variables) or logit regression models (for categorical variables), accounting for region, geographic clustering and various control variables. For analyses of primary outcomes, we present estimates of difference adjusting for geographic clustering (pure), child age and sex (partially adjusted), and also estimates from fully adjusted models controlling for clustering, child age and sex, and maternal, child, and household characteristics that changed significantly over time (those that can affect the outcomes, although they were not directly influenced by the interventions). Logit regression models were used to estimate differences in intake of specific food groups and flesh foods, in order to examine foods specifically promoted by the interventions. We used a one-sided statistical significance level at p-value <0.05 because we hypothesized improvements in the primary outcomes over time. Dose-response analyses using constructed exposure variables of single and multiple intervention channels and analyses by timing of first exposure were conducted using logit regression models (linear regression model for HAZ). To confirm the accuracy of self-reported outcome measures, we measured social desirability to assess for potential biases in our estimates on maternal reported IYCF practices. An adapted five-point social desirability scale measure [28] was developed and tested using logit regression models for differential estimates of the IYCF indicators. Data analysis was performed using Stata 13.

### Ethical approval

This study protocol was approved by the Institutional Review Boards of the Ministry of Science and Technology in Ethiopia and of the International Food Policy Research Institute. Verbal informed consent was obtained from all mothers of study children prior to their participation, given the low literacy of mothers. Completion of the verbal consent procedure was documented by the signature of the interviewer, the date of interview, and the participant’s response at the end of the consent form to indicate that the statements were clearly read aloud and checked for understanding and to record the participant’s response. This verbal consent procedure was approved by the IRBs.

## Results

### Sample characteristics

The distributions of women’s age, religion, underweight, height, index child age and sex, and hygiene score were similar at baseline and endline (Table 1). There were differences in women’s education, occupation, maternal dietary diversity score, body mass index (BMI), low birthweight, number of children less than five years of age, household food insecurity, socioeconomic status, distance to health facility, and at least four antenatal visits. Many of the differences indicate overall improvements in individual and household conditions over time. We accounted for these variables in our fully adjusted models of outcome estimates.

**Table 1:** Sample characteristics by survey round.

Indicator	0–23.9 months		24–59.9 months	
	2010 (n = 1481)	2014 (n = 1494)	2010 (n = 1481)	2014 (n = 1475)
	Mean/Percent	Mean/Percent	Mean/Percent	Mean/Percent
**Maternal characteristics**:				
Age, *years*	28.1 ± 6.2	28.1 ± 6.4	30.2 ± 6.7	30.7 ± 6.9
Education level:				
Never attended school, %	62.5	50.8***	69.8	61.6***
Grade 1–6, %	28.9	32.8	24.8	27.9
Grade 7 or above, %	8.7	16.4***	5.4	10.5***
Occupation as housewife/family farm work, %	93.3	89.6**	93	89.7
Religion as Orthodox Christian, %	40.2	42.6*	41.6	43.6
Dietary diversity score (range 0–9)	2.7 ± 1.5	3.1*** ± 1.5	2.8 ± 1.4	3.1*** ± 1.4
Body mass index, *kg/m*^*2*^	20 ± 2.1	20.3*** ± 2.4	20 ± 2.2	20.2* ± 2.4
Underweight (<18.5 kg/m^2^), %	23.4	23	25.3	24.1
Height, *cm*	157 ± 6.1	157.3 ± 6.1	156.9 ± 5.9	156.8 ± 6
**Child characteristics**:				
Age, *months*	9.2 ± 7	8.9 ± 6.8	38.6 ± 9.9	40.2 ± 9.5
Female, %	49.7	49.4	47.7	50.1
Low birthweight, %	31	26.4*	30.4	22.3***
**Household characteristics**:				
No. of children <5 years	1.6 ± 0.6	1.5*** ± 0.6	1.3 ± 0.5	1.3 ± 0.6
HH food insecurity, %	64.7	51.9***	66.3	53.7***
Hygiene score (range 0–10)	6.5 ± 2.9	6.2 ± 3.1	5.4 ± 3.1	5 ± 3.3
Socioeconomic status (SES)^1^:				
Low, %	42.7	24.4***	42.5	22.6***
Middle, %	33.4	30.9	34.6	33.9
High, %	23.9	44.8***	22.9	43.5***
**Health services access**:				
Distance to health facility, *minutes*	74.9 ± 72	51.2*** ± 72.5	79.8 ± 76.6	49.7*** ± 64.2
At least 4 antenatal visits, %	37.2	51.8***	40.2	48.5**

Significant differences:

***p<0.001,

**p<0.01,

*p<0.05;

p-values obtained from models adjusted for region and clustering effect.

^1^ SES includes house, land and garden ownership, drinking water source, type of toilet, housing material, and cooking fuel type, in addition to household assets.

### Intervention exposure

About 92% of women with children less than two years of age in our sample had been exposed to at least one intervention channel in the past six months (Table 2). Over half of the mothers had been exposed to 2–3 channels, and 19.8% had been exposed to 4 or more channels. With the exception of exposure to the CNCs (72.6%), however, exposure to any other SBCC channels in the six months preceding the endline survey was low (ranging from 8.9% to 31.1%). Only about one-third of mothers had heard any mass media message, 14% had attended a village gathering, and only 8.9% had attended a food demonstration. Overall, contacts with the HEW at the health post and home visit, frequency of contacts, and discussion about IYCF during these contacts had increased significantly between baseline and endline (S1 Table). There were no differences in exposure to SBCC channels by whether or not mothers were exposed to CBN (S2 Table).

**Table 2:** Exposure to program interventions among children 0–23.9 months by age group at endline.

Indicator	2014		
	0–5.9 months (n = 619)	6–23.9 months (n = 875)	0–23.9 months (n = 1494)
	Percent	Percent	Percent
**NO. OF CHANNELS EXPOSED**^1^:			
None	7.8	8.8	8
Low (1 channel)	18.1	22.6	18.2
Medium (2–3 channels)	58.3	52.2	53.9
High (4+ channels)	15.8	16.3	19.8
**Interpersonal communication**:			
Visited health post to discuss IYCF in last 6 months	27.5	22.9	24.8
Received home visit by HEW to discuss IYCF in last 6 months	31.5	26.4	28.5
Received home visit by volunteer to discuss IYCF in last 6 months	12.9	10.1	11.3
Seen Child Nutrition Card (CNC)	73.0	72.3	72.6
**Mass media**:			
Heard any BF radio spot in last 6 months	31.7	30.7	31.1
Heard any CF radio spot in last 6 months	33	29.5	30.9
**Community mobilization**:			
Attended a village gathering about IYCF in last 6 months	11.8	15.6	14
Attended a food demonstration in last 6 months	6.8	10.3	8.9

^1^ Number of intervention channels exposed is based on the number of exposure indicators (range 0-6/7): HEW discussed IYCF during health post visit in last 6 months; HEW discussed IYCF during home visit in last 6 months; volunteer discussed IYCF during home visit in last 6 months; ever seen CNC; heard any BF/CF radio spot in last 6 months; attended village gathering about IYCF in last 6 months, and attended a food demonstration in last 6 months (for CF only).

### Main effects on IYCF practices

There were large and statistically significant increases in the prevalence of all WHO recommended IYCF practices between baseline and endline, except continued BF at one year, which was near universal at baseline (Table 3). These differences remained statistically significant in fully adjusted models. Early initiation of BF increased by 13.7 pp, and EBF increased by 9.4 pp. For CF indicators, the estimates of difference were 22.2, 3.3, 26.2, 3.5, and 2.7 pp for timely introduction of complementary foods, minimum dietary diversity, minimum meal frequency, minimum acceptable diet, and consumption of iron-rich foods, respectively. The prevalence of children with minimum dietary diversity (11.8%), minimum acceptable diet (9.9%), and having consumed iron-rich foods in the past 24 hours (4.8%) remained extremely low at endline.

**Table 3:** IYCF practices by survey round.

Indicator	Age group (months)	2010		2014		Pure^1^ T_2_-T_1_ (pp)	Adjusted^2^ T_2_-T_1_ (pp)	Fully adjusted^3^ T_2_-T_1_ (pp)
		N	Percent	N	Percent			
**Breastfeeding**:								
EIBF (within 1 hour of birth)	0–23.9	1,481	66.7	1,494	81.5	14.8***	14.8***	13.7***
EBF	0–5.9	606	72.4	619	83.4	11.1***	10.2***	9.4***
Continued BF at 1 year	12–15.9	209	98.1	222	95.9	-2.3	-2.6	-1.1
**Complementary feeding**:								
Introduction of solid, semisolid or soft foods	6–8.9	171	37.4	181	59.7	22.5***	21.6***	22.2***
Minimum diet diversity (≥4 food groups)	6–23.9	875	6.3	875	11.8	5.5**	5.8**	3.3*
Minimum meal frequency^4^	6–23.9	875	45.6	875	70.4	24.9***	25.7***	26.2***
Minimum acceptable diet^5^	6–23.9	875	4.6	875	9.9	5.4***	5.6***	3.5*
Consumption of iron-rich food^6^	6–23.9	875	2.3	875	4.8	2.6**	2.8**	2.7**

Significant differences:

***p<0.001,

**p<0.01,

*p<0.05.

^1^ Percentage point difference between baseline and endline adjusted for clustering effect only.

^2^ Percentage point difference between baseline and endline adjusted for clustering effect, child age and sex.

^3^ Percentage point difference between baseline and endline adjusted for clustering effect, child age and sex, and variables with significant differences between baseline and endline.

^4^ Minimum meal frequency is defined as 2 times for breastfed infants 6–8 months; 3 times for breastfed children 9–23 months; and 4 times for non-breastfed children 6–23 months. “Meals” include both meals and snacks, and frequency is based on caregiver report.

^5^ Minimum acceptable diet is defined as having at least the minimum diet diversity and the minimum meal frequency during the previous day.

^6^ Iron-rich or iron-fortified foods include flesh foods; commercially fortified foods especially designed for infants and young children, which contain iron; or foods fortified in the home with a micronutrient powder containing iron.

**Fig 1:**
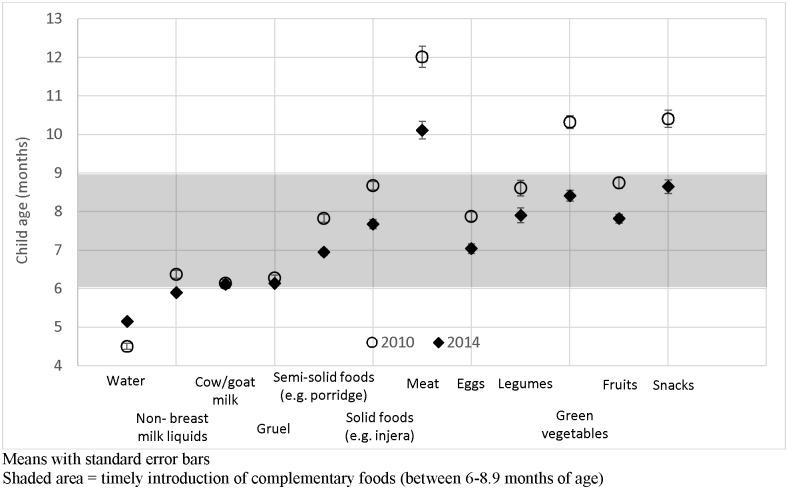
Age of introduction of liquids and foods among children 0–23.9 months by survey round.

There were significant shifts in the age of introduction of various liquids and foods to a more timely introduction between 6 and 8.9 months of age among children less than two years of age (Fig 1). In particular, the mean age of introducing water increased from 4.5 to 5.2 months, thereby shifting closer to the recommended age of introduction. Specific foods promoted by the interventions (i.e., thick porridge, eggs, meat, and green leafy vegetables) also shifted from late introduction to or closer to timely introduction, although semi-solid foods and eggs were already introduced within the appropriate age range at baseline.

We further assessed the individual food groups consumed by children 6–23.9 months of age during the previous 24-hour period, which had increased from 1.7 to 2.1 food groups on average (Table 4). Intake of five out of the seven food groups improved significantly over time, specifically grains (7.1 pp), legumes (13.8 pp), flesh foods (1.8 pp), eggs (7.8 pp), and vitamin-A rich fruits and vegetables (6.5 pp). Intake of dairy products had slightly decreased. Although intake of flesh foods was generally low (3.3%), we examined the disaggregated items and found that meat (beef, pork, lamb, or goat), in particular, had increased significantly (1.5 pp). Intake of specific foods that improved included those promoted by the interventions.

**Table 4:** Intake of food groups in the past 24 hours among children 6–23.9 months by survey round.

Indicator	2010 (n = 875)	2014 (n = 875)	Pure^1^ T_2_-T_1_	Adjusted^2^ T_2_-T_1_	Fully adjusted^3^ T_2_-T_1_
	Mean/ Percent	Mean/ Percent			
No. of food groups	1.7 ± 1.1	2.1 ± 1.2	0.4***	0.4***	0.3***
**Food groups (7)**:					
Grains, roots, and tubers	82.9	88.9	6.1***	7.2***	7.1***
Legumes and nuts	20.2	34.9	15.3***	15.6***	13.8***
Dairy products	32.8	31.0	-2.3	-2.7	-4.0
Flesh foods	1.5	3.3	1.9**	2.1**	1.8*
Eggs	9.6	18.9	9.5***	9.7***	7.8**
Vitamin A-rich fruits and vegetables	20.3	27.0	6.3*	7.5*	6.5*
Other fruits and vegetables	4.1	4.9	0.8	0.9	0.9
**Specific flesh foods**:					
Meat such as beef, pork, lamb, and goat	1.0	2.6	1.6**	1.8**	1.5*
Chicken, ducks, or other poultry	0.2	0.8	0.6	0.6	0.5
Organ meats such as liver, kidney, and heart	0.1	0.7	0.6*	0.6*	0.6*
Fish or shellfish	0.3	0.3	0.0	0.0	0.1

Significant differences:

***p<0.001,

**p<0.01,

*p<0.05.

^1^ Percentage point difference between baseline and endline adjusted for clustering effect only.

^2^ Percentage point difference between baseline and endline adjusted for clustering effect, child age and sex.

^3^ Percentage point difference between baseline and endline adjusted for region, clustering effect, child age and sex, and variables with significant differences between baseline and endline.

There was no evidence of a social desirability bias for any of the IYCF indicators; reported IYCF practices were not associated with social desirability scores (S1 File). Also, we found that IYCF practices did not differ by whether or not mothers were exposed to CBN interventions (S3 Table).

### Main effects on child anthropometry

Although there was a decline from 55.9% to 51.5% in stunting among children 24–59.9 months of age between baseline and endline, the time trend was not significant in the fully adjusted model (Table 5). There were no significant differences in underweight and wasting over time. No significant differences in stunting, underweight, and wasting over time were observed among children 6–23.9 months or 24–35.9 months of age (S4 Table). There were no significant changes in the distribution of HAZ and WAZ over time among the different age groups (S1 Fig; WHZ not shown due to minimal changes).

**Table 5:** Anthropometric indicators of children 24–59.9 months by survey round.

Indicator	2010 (n = 1481)	2014 (n = 1475)	Pure^1^ T_2_-T_1_	Adjusted^2^ T_2_-T_1_	Fully adjusted^3^ T_2_-T_1_
	Mean/Percent	Mean/Percent			
Child stunting	55.9	51.5	-4.4*	-4.3*	-1.5
Child HAZ	-2.1 ± 1.5	-2.1 ± 1.5	0.1	0.1	0.0
Child underweight	30.2	28.4	-1.7	-1.8	0.8
Child WAZ	-1.4 ± 1.2	-1.4 ± 1.2	0.0	0.0	-0.1
Child wasting	4.4	3.8	-0.6	-0.5	-0.4
Child WHZ	-0.2 ± 1.1	-0.2 ± 1.0	0.0	0.0	-0.1

Significant differences:

***p<0.001,

**p<0.01,

*p<0.05.

^1^ Percentage point difference between baseline and endline adjusted for clustering effect only.

^2^ Percentage point difference between baseline and endline adjusted for clustering effect, child age and sex.

^3^ Percentage point difference between baseline and endline adjusted for clustering effect, child age and sex, and variables with significant differences between baseline and endline.

### Child morbidity

There were small but statistically significant declines in ARI symptoms (2.8 pp), and diarrhea (2.6 pp), among children 0–59.9 months, between baseline and endline (S5 Table). Additionally, among children 6–23.9 months, there was a significant decline in ARI symptoms (3.5 pp). In fully adjusted models, there were no other declines in ARI or diarrhea for different age sub-categories.

### Association between intervention exposure and IYCF practices and child growth outcomes: dose-response analysis

We assessed the presence of a dose-response within individual intervention channels and also looked at differential effects on IYCF practices by the number of channels to which mothers were exposed. For individual channels, we found evidence of a dose-response association between increasing numbers of health post visits and early initiation of BF (Table 6). There was also a dose-response relationship for minimum dietary diversity, minimum meal frequency, and minimum acceptable diet and the number of home visits received by volunteer and the number of CNC messages recalled. Specifically, higher numbers of home visits by volunteers (three or more) were associated with 2–2.9 times greater odds of these practices, compared to no home visits; higher numbers of CNC messages recalled (three or more) were associated with 1.8–4.4 greater odds, compared to no CNC messages recalled. Also, higher numbers of CF radio spots heard (three or more) were associated with 3.1 and 2.9 greater odds of minimum dietary diversity and minimum acceptable diet, respectively, compared to no recall of CF radio spots. These findings suggest a plausible association between the program interventions and improvements in early initiation of BF, minimum dietary diversity, minimum meal frequency, and minimum acceptable between 2010 and 2014 in study areas. Contrary to expectation, exposure to a BF radio spot was associated with lower odds of early initiation of the practice. Also, there was no dose-response association between exposure to SBCC channels and EBF.

**Table 6:** Association between intervention intensity or multiple channels exposure and IYCF practices^1^.

	EIBF (0–23.9 mos) (n = 1481)	EBF (0–5.9 mos) (n = 606)	Minimum dietary diversity (6–23.9 mos) (n = 875)	Minimum meal frequency (6–23.9 mos) (n = 875)	Minimum acceptable diet (6–23.9 mos) (n = 875)	Consumption of iron-rich foodsv (6–23.9 mos) (n = 875)
Variables	OR	OR	OR	OR	OR	OR
**Intensity of individual intervention channels (IPC and mass media)**						
No. of health post visits in last 6 months:						
None	Ref	Ref	Ref	Ref	Ref	Ref
1–2 visits	1.53*	0.68	1.58	0.97	1.33	1.28
3+ visits	1.81**	0.68	2.16	1.58	1.95	1.25
No. of home visits by HEW in last 6 months:						
None	Ref	Ref	Ref	Ref	Ref	Ref
1–2 visits	1.40	0.70	0.72	0.96	0.67	0.91
3+ visits	1.62	0.94	1.56	1.19	1.49	1.35
No. of home visits by volunteer in last 6 months:						
None	Ref	Ref	Ref	Ref	Ref	Ref
1–2 visits	1.36	1.20	1.73	1.07	1.52	1.46
3+ visits	1.31	1.56	2.53*	2.01*	2.92*	1.68
No. of CNC messages recalled:						
None	Ref	Ref	Ref	Ref	Ref	Ref
1 BF/1-2 CF messages	1.26	0.85	1.58	0.87	1.49	2.78
2 BF/3-5 CF messages	1.49	2.00	4.35***	1.75*	3.85***	2.72
No. of radio spots heard:						
None	Ref	Ref	Ref	Ref	Ref	Ref
1 BF/1-2 CF spots	0.70*	1.21	2.62*	1.07	2.06	1.26
2 BF/3-4 CF spots	1.10	0.71	3.07***	1.06	2.88**	1.32
**NO. OF CHANNELS EXPOSED**^2^:						
None	Ref	Ref	Ref	Ref	Ref	Ref
Low (1 channel)	1.88*	1.51	6.15	1.06	5.20	1.50
Medium (2–3 channels)	2.07*	1.39	6.87*	1.31	5.82	1.72
High (4+ channels)	2.48*	0.93	18.75**	2.04	14.19**	2.53

Significant differences:

***p<0.001,

**p<0.01,

*p<0.05.

^1^ Multivariate model adjusted for child factors (age, sex, and birthweight), maternal factors (age, education, occupation, religion), and household factors (number of children <5y, SES, food insecurity, and distance to health facility).

^2^ Number of intervention channels exposed is based on the number of exposure indicators (range 0-6/7): HEW discussed IYCF during health post visit in last 6 months; HEW discussed IYCF during home visit in last 6 months; volunteer discussed IYCF during home visit in last 6 months; ever seen CNC; heard any BF/CF radio spot in last 6 months; attended village gathering about IYCF in last 6 months, and attended a food demonstration in last 6 months (for CF only).

With respect to the number of channels to which mothers were exposed, we observed a strong dose-response association between exposure to increasing SBCC channels and early initiation of BF, minimum dietary diversity, and minimum acceptable diet (Table 6). There was no clear dose-response association between exposure to SBCC channels and child stunting or HAZ (Table 7). The timing of first intervention exposure was also not statistically significantly associated with stunting or HAZ.

**Table 7:** Association between exposure to multiple channels or timing of first intervention exposure and stunting and HAZ among children 24–59.9 months^1^.

	Stunting (24–59.9 months) (n = 1481)	HAZ (24–59.9 months) (n = 1475)
Variables	OR	β
**NO. OF CHANNELS EXPOSED**^2^:		
None	Ref	Ref
Low (1 channel)	1.11	0.97
Medium (2–3 channels)	0.91	1.04
High (4+ channels)	1.20	0.84
**TIMING OF FIRST EXPOSURE**^3^:		
Too late/no exposure (≥24 months)	Ref	Ref
Late exposure (12–23.9 months)	0.57	1.67
Early exposure (0–11.9 months)	0.71	1.36
Optimal timing of exposure (pregnancy to birth)	1.50	1.21

Significant differences:

***p<0.001,

**p<0.01,

*p<0.05.

^1^ Multivariate models adjusted for child factors (age, age squared, sex, ARI, diarrhea, and birthweight), maternal factors (age, height, education, occupation, religion), and household factors (number of children <5y, SES, food insecurity, distance to health facility).

^2^ Number of intervention channels exposed is based on the number of exposure indicators (range 0–7): HEW discussed IYCF during health post visit in last 6 months; HEW discussed IYCF during home visit in last 6 months; volunteer discussed IYCF during home visit in last 6 months; ever seen CNC; heard any BF/CF radio spot in last 6 months; attended village gathering about IYCF in last 6 months, and attended a food demonstration in last 6 months.

^3^ Timing of first intervention exposure is based on the estimation of child age at first exposure (i.e., child age at endline–implementation duration, starting at January 1, 2012):

Too late/no exposure = likely unexposed to the program because child age ≥24 months at first exposure

Late exposure = first potential exposure at child age 12–23.9 months

Early exposure = first potential exposure at child age 0–11.9 months

Optimal timing of exposure = first potential exposure is before child birth (during pregnancy)

## Discussion

Exposure to large-scale SBCC interventions was associated with large improvements in BF and CF practices in the two food-insecure regions (SNNPR and Tigray) included in this evaluation of A&T in Ethiopia. In particular, the behaviors and intake of foods promoted by the interventions improved markedly. Our findings are in line with evidence from an earlier adequacy evaluation of the CBN program, of improved EBF and CF practices after about 28 months of exposure to counseling of mothers and monthly community conversations [27]. We did not see any differences in IYCF practices between mothers exposed to CBN compared to those not exposed in our study population.

Despite significant increases in the IYCF indicators, several CF practices remain very poor. At endline, minimum dietary diversity was low at 11.8%, minimum acceptable diet was 9.9%, and consumption of iron-rich food was 4.8%. Although the mean number of food groups had increased from 1.7 to 2.1 (out of seven food groups) from 2010 to 2014, this still indicates intake of poor quality diets among young children and an extremely low probability of children meeting their micronutrient (and probably also energy) requirements. CF indicators in our study such as minimum dietary diversity, for instance, are higher than the country average (4.8%) or even regional averages (3.8% in SNNPR and 6.0% in Tigray) for 2011 [5], but results from the 2013 National Food Consumption Survey and 2015 National Nutrition Program Endline Survey also show highly monotonous diets consisting of mostly cereals/grains and diet deficient in energy and insufficient nutrient density consumed by young children [29] with little to no improvements in CF practices between 2009 and 2015 [30]. Given our understanding that household food insecurity is associated with dietary diversity [31,32] and limited food availability and resources may constrain adoption of some recommended child feeding practices, the large remaining gaps in CF practices need to be improved and sustained in this population by addressing food insecurity and other constraints in addition to implementing effective SBCC strategies. While household food security improved over time in our study population, more than half of the households were still food-insecure, and with the persistent food security challenges in the country [33,34], the important role of household food security and enabling conditions for adoption of recommended practices need to be considered in Ethiopia.

We did not find a statistically significant decline in child stunting over time in our study areas. The magnitude of the decline in our study (4.4 pp) was similar to stunting declines reported for children less than five years of age between 2011 and 2014 (decline of 4 pp, from 44% to 40%) nationally reported in the 2011 Ethiopia Demographic and Health Survey (DHS) [6] and 2014 Mini-DHS [3]). This suggests that the changes in child growth were likely due to secular patterns experienced in the whole country over the study period. This inference is further supported by the positive changes observed in our sample in household socioeconomic status and food security, maternal education and anthropometry, and the percentage of children with adequate birth weight, which suggest significant improvements in several of the underling determinants of child anthropometry. The Mini-DHS [3] also documents increased access to antenatal care and other maternal care indicators in the same regions. Furthermore, preliminary results from the 2014 Productive Safety Net Program survey in the highland areas, which includes Tigray and SNNPR, indicated similar trends in some child-level and household factors such as improved household socioeconomic status and access to health and basic services, and similar stunting prevalence among children 24–59.9 months (N. Kumar and J. Hoddinott, personal communication, December 4, 2015). Thus, accelerating improvements in the nutritional status of children in Ethiopia likely requires a series of additional inputs from multiple sectors, including improving IYCF awareness, knowledge, and practices, as well as other strategies.

Overall exposure to the A&T interventions was relatively low, and the challenges of achieving reach into the communities and target households are discussed in a separate paper [18]. In addition to the gap identified between the formal health system (health extension program) and the community volunteers in the delivery chain, HEWs’ work context such as work priorities, time, and workload were suggested as potential constraints in our evaluation and in other studies [35,36]. Extensive formative research was conducted by A&T prior to implementation to develop the comprehensive and context-specific communication strategies and messages [37], and the reach of the strategies was monitored through program monitoring and process and impact evaluations. While moderate exposure was anticipated and various strategies were designed to reinforce the reach as well as the messages, actual exposure to interventions were lower than expected. In Ethiopia, coverage of interventions, even basic health services such as full immunization for young children is low (24.3% [5]), and remains a challenge that needs to be continually addressed and overcome through improved delivery systems.

Despite low exposure to the interventions, however, the plausibility of effect of these interventions on IYCF practices is supported by our findings from dose-response analyses. Several IYCF practices were associated with higher intensity of exposure to individual intervention channels (i.e., increasing numbers of health post visits and early initiation of BF, and higher numbers of home visits by volunteers, higher numbers of CNC messages recalled, and higher numbers of CF radio spots heard with greater odds of minimum dietary diversity, minimum meal frequency, and minimum acceptable diet) as well as to exposure to a larger number of SBCC channels. While most intervention channels are distinct and distinguishable to respondents, some may have been interlinked in the delivery process. For example, HEWs and volunteers usually conduct home visits separately, but there may have been instances when they visited a home together, particularly for supervision or handling of difficult cases that require special attention. There may also have been overlap in seeing the CNC and home visits by HEWs or volunteers, who may use the CNC as a counseling aid. However, the potential double-counting of exposure to intervention channels is unlikely to have made a difference in the dose-response associations based on increasing number of exposure to specific channels, and associations between IYCF practices and the number of SBCC channels were significant mostly for high exposure to four or more channels. Our results concur with findings from a recent review that suggests that a combination of interventions (i.e., education and support at the individual and groups levels) delivered through different platforms such as the health system and community-based activities are more effective at improving BF practices than a single intervention [9].

There are several limitations to our study. First, the adequacy design does not allow us to infer causality or attribution of changes in outcomes to the program. Our plausibility analyses, however, support the argument that changes observed in several of the IYCF practices were associated with intervention exposure. Furthermore, in cluster-randomized program evaluations of the same types of interventions–counseling, community mobilization and mass media–conducted as part of the impact evaluation of the A&T initiative in other countries [38,39], we found similar results: impacts on feeding practices but not on growth outcomes. Second, our repeated cross-sectional design estimated changes in IYCF practices and child anthropometry between 2010 and 2014, assessing different children of the same age; our study did not include a longitudinal component that would have allowed documenting changes over time in a cohort of children. However, in the assessment of changes in practices in the context of large-scale programs, it is not feasible to assess impacts on individual children given longer range timelines required to achieve implementation quality and strengthen coverage. The current design was the most feasible given the size and context of the intervention program in Ethiopia. Third, results on IYCF practices are based on maternal recall data using standard methods [24]. Although recall data could have been influenced by social desirability, our analysis for the role of social desirability [28] indicated no bias. Fourth, we did not measure the intake of food quantity among children. While food quantity is an important dimension of complementary feeding practices and may have contributed to the poor nutritional status of children, quantity was not a key message of the interventions and not assessed as a main outcome. Given the extremely low proportion of children achieving minimum acceptable diet, we expect very poor nutrient intake in our study population, and data on estimated portion size of food consumed would likely have corroborated these results. Lastly, the study sample was drawn only from the Integrated Family Health Program woredas in two regions; thus our results are not representative of the overall program.

## Conclusions

Exposure to the A&T’s SBCC interventions in Ethiopia was significantly and plausibly associated with improved IYCF practices. Larger effects might have been achieved with greater coverage and higher intensity. While improvements were achieved across various IYCF practices, there remains a large unfinished agenda for improving children’s diets and nutritional status, particularly in the complementary feeding period, by addressing household food insecurity and other constraints to enable conditions for adoption of recommended practices promoted by effective SBCC strategies in Ethiopia.

## Supporting Information

S1 Fig Distributions of HAZ and WAZ by age group and survey round. (TIFF)

S1 File Social desirability bias. (DOCX)

S1 Table Exposure to HEWs and community volunteers among children 0–23.9 months by survey round. (DOCX)

S2 Table Exposure and intensity of interventions among children 0–23.9 months by CBN exposure at endline. (DOCX)

S3 Table IYCF practices by CBN exposure at endline. (DOCX)

S4 Table Anthropometric indicators for children 6–23.9 and 24–35.9 months by survey round. (DOCX)

S5 Table Prevalence of ARI symptoms and diarrhea among children 0–59.9 months by age group and survey round. (DOCX)
