# Supplementary material for: Exposure to Large-Scale Social and Behavior Change Communication Interventions Is Associated with Improvements in Infant and Young Child Feeding Practices in Ethiopia
Source: PLoS One. 2016 Oct 18;11(10):e0164800. doi: 10.1371/journal.pone.0164800 (PMC5068829; doi:10.1371/journal.pone.0164800)
Supplement: S1 File — (DOCX) [file pone.0164800.s002.docx]

**S1 File. Social desirability bias**

Bivariate analyses between IYCF practices and social desirability score (SDS, range 0-5) as two-level categories (0-2 as low and 3-5 as high) showed no significant difference in IYCF practices between the SDS categories (Table 1). Using a four-level SDS category (0-2 as low, 3 as medium, 4 as high, and 5 as very high), there was a significant association between early initiation of breastfeeding and very high SDS only (Table 2).

**Table 1. IYCF practices among those with low or high SDS (2 categories)**

| **SDS**  **category** | **EIBF** | **EBF** | **Cont. BF** | **Timely intro** | **Min diet diversity** | **Min frequency** | **Min acc. diet** | **Cons. Fe-rich** |
| --- | --- | --- | --- | --- | --- | --- | --- | --- |
|  | **N=1494** | **N=619** | **N=222** | **N=181** | **N=875** | **N=875** | **N=875** | **N=875** |
|  | **Percent** | **Percent** | **Percent** | **Percent** | **Percent** | **Percent** | **Percent** | **Percent** |
| Low (<=2) | 77.8 | 75.8 | 96.3 | 72 | 16.2 | 70.5 | 12.4 | 4.8 |
| High (>=3) | 82.0 | 84.2 | 95.9 | 57.7 | 11.0 | 70.4 | 9.4 | 3.8 |
| P-value | 0.143 | 0.12 | 0.892 | 0.207 | 0.155 | 0.985 | 0.323 | 0.669 |

Significant differences: *p<0.05

**Table 2. IYCF practices among those with low to high SDS (4 categories)**

| **SDS category** | **EIBF** | **EBF** | **Cont. BF** | **Timely intro** | **Min diet diversity** | **Min frequency** | **Min acc. diet** | **Cons. Fe-rich** |
| --- | --- | --- | --- | --- | --- | --- | --- | --- |
|  | **N=1494** | **N=619** | **N=222** | **N=181** | **N=875** | **N=875** | **N=875** | **N=875** |
|  | **Percent** | **Percent** | **Percent** | **Percent** | **Percent** | **Percent** | **Percent** | **Percent** |
| Low (0-2) | 77.8 | 75.8 | 96.3 | 72 | 16.2 | 70.5 | 12.4 | 4.8 |
| Medium (3) | 76.2 | 82.1 | 93.6 | 51.1 | 10.6 | 67.2 | 8.1 | 3.5 |
| High (4) | 81.0 | 80.8 | 94.6 | 55.6 | 10.3 | 67.6 | 8.9 | 4.7 |
| Very high (5) | **85.5*** | 86.9 | 97.8 | 63.6 | 11.7 | 73.9 | 10.5 | 3.4 |
| P-value | 0.020 | 0.105 | 0.355 | 0.258 | 0.537 | 0.302 | 0.649 | 0.820 |

Significant differences: *p<0.05

However, there was no association between SDS and IYCF practices, nor differential effects by region, based on regression analyses (Table 3).

**Table 3. Association between IYCF practices and SDS**

| **Variable** | **EIBF** | **EBF** | **Cont. BF** | **Timely intro** | **Min diet diversity** | **Min frequency** | **Min acc. diet** | **Cons. Fe-rich** |
| --- | --- | --- | --- | --- | --- | --- | --- | --- |
|  | **N=1494** | **N=619** | **N=222** | **N=181** | **N=875** | **N=875** | **N=875** | **N=875** |
| SDS (range 0-5) | 0.110 | 0.046 | -1.022 | -0.316 | -0.176 | -0.059 | -0.095 | 0.120 |
|  | (0.319) | (0.755) | (0.276) | (0.267) | (0.312) | (0.635) | (0.608) | (0.549) |
| SNNP Region | -0.308 | -0.299 | -6.058 | -1.987 | -0.625 | -0.253 | -0.577 | -0.371 |
|  | (0.593) | (0.719) | (0.198) | (0.129) | (0.467) | (0.683) | (0.531) | (0.769) |
| SNNPR*SDS | 0.094 | 0.245 | 2.261 | 0.461 | 0.125 | 0.128 | 0.130 | -0.302 |
|  | (0.495) | (0.227) | (0.062) | (0.168) | (0.557) | (0.402) | (0.569) | (0.344) |
| Constant | 1.216 | 1.102 | 8.550 | 1.756 | -1.523 | 0.996 | -2.018 | -2.882 |
|  | (0.008) | (0.068) | (0.068) | (0.110) | (0.024) | (0.048) | (0.005) | (0.000) |
| Observations | 1485 | 617 | 220 | 181 | 868 | 868 | 868 | 868 |
| EA/clusters | 75 | 75 | 69 | 68 | 75 | 75 | 75 | 75 |

Significant differences: *p<0.05
