# Supplementary material for: Exposure to Large-Scale Social and Behavior Change Communication Interventions Is Associated with Improvements in Infant and Young Child Feeding Practices in Ethiopia
Source: PLoS One. 2016 Oct 18;11(10):e0164800. doi: 10.1371/journal.pone.0164800 (PMC5068829; doi:10.1371/journal.pone.0164800)
Supplement: S1 Table — (DOCX) [file pone.0164800.s003.docx]

**S1 Table. Exposure to HEWs and community volunteers among children 0-23.9 months by survey round**

| **Indicator** | **0-5.9 months** | | **6-23.9 months** | |
| --- | --- | --- | --- | --- |
|  | **2010** | **2014** | **2010** | **2014** |
|  | **(n=606)** | **(n=619)** | **(n=875)** | **(n=875)** |
|  | **Percent** | **Percent** | **Percent** | **Percent** |
| Visited the health post in last 6 months | 51 | 73.7*** | 46.9 | 65.3*** |
| None | 49.4 | 26.4*** | 54.6 | 34.9*** |
| 1-2 health post visits | 29.3 | 36.6* | 26.4 | 40.3*** |
| 3+ health post visits | 21.3 | 37.0*** | 19.1 | 24.9* |
| HEW discussed IYCF during health post visit in last 6 months | 10.6 | 27.5*** | 14.9 | 22.9*** |
| Received home visit by HEW in last 6 months | 33.4 | 49.8*** | 31 | 40.8** |
| None | 67.2 | 50.3*** | 69 | 59.7** |
| 1-2 HEW visits | 21.5 | 30.6** | 16.9 | 22.1* |
| 3+ HEW visits | 11.3 | 19.1** | 14.1 | 18.2 |
| HEW discussed IYCF during home visit in last 6 months | 15.1 | 31.5*** | 10.4 | 26.4*** |
| Received home visit by volunteer in last 6 months | 35.3 | 32.4 | 32 | 29.9 |
| None | 65.6 | 68.3 | 68.8 | 70.8 |
| 1-2 volunteer visits | 20.9 | 18.1 | 17.7 | 13.6 |
| 3+ volunteer visits | 13.6 | 13.6 | 13.4 | 15.6 |
| Volunteer discussed IYCF during home visit in last 6 months | 9 | 12.9 | 7.1 | 10.1* |

Significant differences: ***p<0.001, **p<0.01, *p<0.05; p-values obtained from models adjusted for clustering effect.
