# Supplementary material for: Exposure to Large-Scale Social and Behavior Change Communication Interventions Is Associated with Improvements in Infant and Young Child Feeding Practices in Ethiopia
Source: PLoS One. 2016 Oct 18;11(10):e0164800. doi: 10.1371/journal.pone.0164800 (PMC5068829; doi:10.1371/journal.pone.0164800)
Supplement: S2 Table — (DOCX) [file pone.0164800.s004.docx]

**S2 Table. Exposure and intensity of interventions among children 0-23.9 months by CBN exposure at endline**

| **Indicator** | **2014** | | | | |
| --- | --- | --- | --- | --- | --- |
|  | **CBN exposure** | | | **No CBN exposure** | |
|  | **N** | **Percent** | **N** | | **Percent** |
| **Interpersonal communication** |  |  |  | |  |
| Received home visit by HEW in last 6 months: | 414 | 44.2 | 241 | | 45.1 |
| No HEW visits | 522 | 56.0 | 295 | | 55.6 |
| 1-2 HEW visits | 254 | 27.2 | 121 | | 22.8 |
| 3+ HEW visits | 157 | 16.8 | 115 | | 21.7 |
| HEW talked about IYCF during a home visit in last 6 months | 275 | 29.4 | 143 | | 26.8 |
| Received home visit by community volunteer in last 6 months: | 291 | 31.2 | 165 | | 30.9 |
| No volunteer visits | 642 | 69.4 | 369 | | 69.9 |
| 1-2 volunteer visits | 147 | 15.9 | 78 | | 14.8 |
| 3+ volunteer visits | 136 | 14.7 | 81 | | 15.3 |
| Volunteer talked about IYCF during a home visit in last 6 months | 103 | 11.1 | 65 | | 12.2 |
| Ever seen CNC/Tool B | 673 | 72.0 | 396 | | 74.0 |
| CNC messages recalled: |  |  |  | |  |
| No BF messages | 689 | 73.7 | 393 | | 73.5 |
| 1 BF message | 160 | 17.1 | 89 | | 16.6 |
| 2 BF messages | 86 | 9.2 | 53 | | 9.9 |
| No CF messages | 372 | 39.7 | 228 | | 42.7 |
| 1-2 CF messages | 347 | 37.1 | 193 | | 36.1 |
| 3-5 CF messages | 217 | 23.2 | 113 | | 21.2 |
| **Mass media** |  |  |  | |  |
| Heard any BF radio spot in last 6 months | 304 | 32.4 | 150 | | 28.0 |
| BF radio spots heard: |  |  |  | |  |
| No BF radio spots heard | 633 | 67.6 | 385 | | 72.0 |
| 1 radio spot heard | 147 | 15.7 | 60 | | 11.2 |
| 2 radio spots heard | 157 | 16.8 | 90 | | 16.8 |
| BF radio spot messages recalled: |  |  |  | |  |
| No BF radio message recalled | 808 | 86.2 | 468 | | 87.5 |
| 1 radio message recalled | 81 | 8.6 | 28 | | 5.2 |
| 2 radio messages recalled | 48 | 5.1 | 39 | | 7.3 |
| Heard any CF radio spot in last 6 months | 312 | 33.3 | 140 | | 26.2 |
| CF radio spots heard: |  |  |  | |  |
| No CF radio spots heard | 624 | 66.7 | 395 | | 73.8 |
| 1-2 CF radio spots heard | 85 | 9.1 | 28 | | 5.2 |
| 3-4 CF radio spots heard | 72 | 7.7 | 33 | | 6.2 |
| CF radio spot messages recalled: |  |  |  | |  |
| No CF radio message recalled | 759 | 81.1 | 443 | | 82.8 |
| 1-2 CF radio messages recalled | 71 | 7.6 | 42 | | 7.9 |
| 3-4 CF radio messages recalled | 52 | 5.6 | 21 | | 3.9 |
| **Community mobilization** |  |  |  | |  |
| Attended a food demonstration in last 6 months | 81 | 8.7 | 51 | | 9.5 |
| Attended a village gathering on IYCF in last 6 months | 136 | 14.6 | 71 | | 13.3 |
| **NO. OF CHANNELS EXPOSED^1^:** |  |  |  | |  |
| **Related to breastfeeding** |  |  |  | |  |
| None | 163 | 17.4 | 85 | | 15.9 |
| Low (1 channel) | 322 | 34.4 | 218 | | 40.8 |
| Medium (2-3 channels) | 383 | 40.9 | 193 | | 36.1 |
| High (4+ channels) | 69 | 7.4 | 39 | | 7.3 |
| **Related to complementary feeding** |  |  |  | |  |
| None | 158 | 16.9 | 90 | | 16.8 |
| Low (1 channel) | 319 | 34.0 | 218 | | 40.8 |
| Medium (2-3 channels) | 389 | 41.5 | 189 | | 35.3 |
| High (4+ channels) | 71 | 7.6 | 38 | | 7.1 |

Significant differences: *p<0.05; p-values obtained from models adjusted for clustering effect.

^1^ Number of intervention channels exposed is based on the number of program exposure indicators (range 0-6/7): HEW discussed IYCF during health post visit in last 6 months; HEW discussed IYCF during home visit in last 6 months; volunteer discussed IYCF during home visit in last 6 months; ever seen CNC; heard any BF/CF radio spot in last 6 months; attended village gathering about IYCF in last 6 months, and attended a food demonstration in last 6 months (for CF only).
