# Supplementary material for: Exposure to Large-Scale Social and Behavior Change Communication Interventions Is Associated with Improvements in Infant and Young Child Feeding Practices in Ethiopia
Source: PLoS One. 2016 Oct 18;11(10):e0164800. doi: 10.1371/journal.pone.0164800 (PMC5068829; doi:10.1371/journal.pone.0164800)
Supplement: S3 Table — (DOCX) [file pone.0164800.s005.docx]

**S3 Table. IYCF practices by CBN exposure at endline**

| **Indicator** | **Age group** | **2014** | | | |
| --- | --- | --- | --- | --- | --- |
|  |  | **CBN exposure** | | **No CBN exposure** | |
|  | **Months** | **N** | **Percent** | **N** | **Percent** |
| Early initiation of breastfeeding | 0-23.9 | 756 | 80.7 | 448 | 83.7 |
| Exclusive breastfeeding | 0-5.9 | 322 | 83.6 | 188 | 83.6 |
| Continued breastfeeding at 1 year | 12-15.9 | 154 | 95.1 | 56 | 98.3 |
| Introduction of solid, semi-solid, or soft foods | 6-8.9 | 66 | 66.0 | 42 | 51.9 |
| Minimum dietary diversity | 6-23.9 | 73 | 13.2 | 27 | 8.7 |
| Minimum meal frequency | 6-23.9 | 402 | 72.8 | 205 | 66.1 |
| Minimum acceptable diet | 6-23.9 | 60 | 10.9 | 25 | 8.1 |
| Consumption of iron rich foods | 6-23.9 | 25 | 4.5 | 11 | 3.6 |

Significant differences: *p<0.05; p-values obtained from models adjusted for clustering effect.
