# Supplementary material for: Exposure to Large-Scale Social and Behavior Change Communication Interventions Is Associated with Improvements in Infant and Young Child Feeding Practices in Ethiopia
Source: PLoS One. 2016 Oct 18;11(10):e0164800. doi: 10.1371/journal.pone.0164800 (PMC5068829; doi:10.1371/journal.pone.0164800)
Supplement: S4 Table — (DOCX) [file pone.0164800.s006.docx]

**S4 Table. Anthropometric indicators for children 6-23.9 and 24-35.9 months by survey round**

| **Indicator** | **2010** | **2014** | **Pure^1^**  **T_2_-T_1_** | **Adjusted^2^ T_2_-T_1_** | **Fully adjusted^3^ T_2_-T_1_** |
| --- | --- | --- | --- | --- | --- |
|  | **(n=1481)** | **(n=1475)** |  |  |  |
|  | **Mean/Percent** | **Mean/Percent** |  |  |  |
| **6-23.9 months** |  |  |  |  |  |
| Child stunting | 41.8 | 40.7 | -1.0 | 0.2 | 2.0 |
| Child HAZ | -1.7 ± 1.6 | -1.5 ± 1.7 | 0.1 | 0.1 | 0.1 |
| Child underweight | 23 | 24 | 1.3 | 1.4 | 3.4 |
| Child WAZ | -1.2 ± 1.3 | -1.1 ± 1.3 | 0.0 | 0.0 | -0.1 |
| Child wasting | 8.5 | 7.6 | -0.8 | -1.0 | -0.3 |
| Child WHZ | -0.4 ± 1.3 | -0.4 ± 1.2 | 0.0 | 0.0 | -0.1 |
| **24-35.9 months** |  |  |  |  |  |
| Child stunting | 55.1 | 53.6 | -1.6 | -1.4 | 0.4 |
| Child HAZ | -2.2 ± 1.5 | -2 ± 1.6 | 0.1 | 0.2 | 0.1 |
| Child underweight | 32.1 | 28.8 | -3.6 | -3.8 | -3.2 |
| Child WAZ | -1.4 ± 1.3 | -1.3 ± 1.2 | 0.1 | 0.1 | 0.0 |
| Child wasting | 5.5 | 4.8 | -0.7 | -0.7 | -0.4 |
| Child WHZ | -0.2 ± 1.1 | -0.2 ± 1.1 | 0.0 | 0.0 | 0.0 |

Significant differences: ***p<0.001, **p<0.01, *p<0.05

**^1^** Percentage point difference between baseline and endline adjusted for clustering effect only

**^2^** Percentage point difference between baseline and endline adjusted for clustering effect, child age and sex.

**^3^** Percentage point difference between baseline and endline adjusted for clustering effect, child age and sex, and variables with significant differences between baseline and endline.
