# Supplementary material for: Exposure to Large-Scale Social and Behavior Change Communication Interventions Is Associated with Improvements in Infant and Young Child Feeding Practices in Ethiopia
Source: PLoS One. 2016 Oct 18;11(10):e0164800. doi: 10.1371/journal.pone.0164800 (PMC5068829; doi:10.1371/journal.pone.0164800)
Supplement: S5 Table — (DOCX) [file pone.0164800.s007.docx]

**S5 Table. Prevalence of ARI symptoms and diarrhea among children 0-59.9 months by age group and survey round**

| **Indicators/age group** | **2010** | | **2014** | | | | **Pure^1^**  **T_2_-T_1_** | **Adjusted^2^ T_2_-T_1_** | **Fully adjusted^3^ T_2_-T_1_** |
| --- | --- | --- | --- | --- | --- | --- | --- | --- | --- |
|  | **N** | **Percent** | **N** | | **Percent** | |  |  |  |
| **ARI symptoms in past 2 weeks** | | | | | | | | | |
| 0–5.9 months | 606 | 8.3 | | 619 | | 4.7 | -3.5* | -3.6* | -2.1 |
| 6-23.9 months | 875 | 10.3 | | 875 | | 5.9 | -4.4** | -4.3** | -3.5* |
| 24-59.9 months | 1481 | 7.4 | | 1475 | | 3.4 | -4** | -3.7** | -2.6 |
| 0-59.9 months | 2962 | 8.5 | | 2969 | | 4.4 | -4.1*** | -3.9*** | -2.8* |
| **Diarrhea in past 2 weeks** | | | | | | | | | |
| 0–5.9 months | 606 | 14.6 | | 619 | | 9.9 | -4.6* | -4.1 | -3.2 |
| 6-23.9 months | 875 | 22.4 | | 875 | | 17.9 | -4.5* | -4.6* | -2.7 |
| 24-59.9 months | 1481 | 12.6 | | 1475 | | 8.1 | -4.5** | -3.8** | -1.9 |
| 0-59.9 months | 2962 | 15.9 | | 2969 | | 11.4 | -4.5*** | -4.2*** | -2.6* |

Significant differences: ***p<0.001, **p<0.01, *p<0.05

**^1^** Percentage point difference between baseline and endline adjusted for clustering effect only

**^2^** Percentage point difference between baseline and endline adjusted for clustering effect, child age and sex.

**^3^** Percentage point difference between baseline and endline adjusted for clustering effect, child age and sex, and variables with significant differences between baseline and endline.
